# An international survey on canine urinary incontinence: case frequency, diagnosis, treatment and follow-up

**DOI:** 10.3389/fvets.2024.1360288

**Published:** 2024-07-17

**Authors:** M. V. Falceto, R. Caccamo, A. M. Garrido, M. C. Pisu, M. T. Tejedor, P. Trerotoli, S. Nicoli, P. Zagarella, I. Lippi, E. García-Pedraza, J. Rambaldi, D. Kirilova, O. Mitjana

**Affiliations:** ^1^Department of Animal Pathology, Agroalimentary Institute of Aragon-IA2, Universidad de Zaragoza-CITA, Zaragoza, Spain; ^2^Dipartimento di Scienze Veterinarie, Universitá di Torino, Torino, Italy; ^3^Centro Referenza Veterinario, Torino, Italy; ^4^Department of Anatomy, Embriology and Animal Genetics, Ciber CV, Universidad de Zaragoza, Zaragoza, Spain; ^5^Dipartimento Interdisciplinare di Medicina, Universitá degli Studi di Bari Aldo Moro, Bari, Italy; ^6^AniCura, Clinica Veterinaria Roma Sud, Rome, Italy; ^7^CTO Veterinario S.r.l., Arenzano, Italy; ^8^Dipartimento di Scienze Veterinarie, Università di Pisa (PI), Pisa, Italy; ^9^Vetoquinol, Paris, France

**Keywords:** urinary incontinence, USMI, dog, questionnaire, recommendations

## Abstract

**Introduction:**

Urinary incontinence (UI) consists of involuntary leakage of urine during the storage phase of urination.

**Methods:**

An anonymous survey was given to Spanish and Italian veterinarians about canine UI treated cases, diagnosis, treatment, follow-up, and professional interest.

**Results and discussion:**

Most veterinarians treated ≤3 cases/quarter, resulting in the percentage of incontinence males being lower than that of females (1-4% vs 0-24%). The percentage of spayed incontinent females was lower in Spain (0-24%) than in Italy (75-100%). Most diagnoses were based on a diagnostic algorithm (Spain: 88.7%; Italy: 65.3%); patient report and history, blood work, urinalysis and abdominal ultrasound. Urethral/bladder pressure measurement was unusual (Spain: 0.2%; Italy: 2.4%). In Spain, radiology with contrast medium and CT urography (26.3% and 34.4%, respectively) were more frequent than in Italy (11.6% and 22.7%, respectively). When suspecting urethral sphincter mechanism incompetence pharmacological trial (Spain: 93.2%; Italy: 78.9%). The first-choice medical treatment was Phenylpropanolamine, followed by Ephedrine and Deslorelin. When pharmacotherapy failed, the most frequent option was drug change, followed by increased drug dosage/frequency of administration, surgical therapy and colposuspension. A review was completed after the first week of treatment followed by periodic reviews. Most of the respondents participated in continuing education only if UI occurred in their everyday practice (Spain: 63.0%; Italy: 55.4%) and about 30% responders did it regardless of the number of UI cases treated (Spain: 30.5%; Italy: 37.4%).

**Conclusion:**

Some recommendations in clinical practice were made. UI can be underestimated by owners; therefore, a complete history should be obtained by veterinarians. Veterinarians should carefully evaluate if spaying is advisable considering it could increase UI risk. A step-by-step approach is recommended and a specific diagnostic-therapeutic algorithm for UI in dogs is provided. Conservative approaches (regular exercise, weight loss in overweight dogs and observing an “incontinence diary” to identify abnormal patterns of urination) are advisable.

## Introduction

Urinary incontinence (UI) is defined as the involuntary leakage of urine during the storage phase of urination (1, 2). Neutering, breed, body size, tail-cutting, obesity and age were described as risk factors for UI, considered as a complex and multifactorial condition (3, 4).

Usually, UI occurs in puppies (<6 months) as a congenital form or in adults as an acquired form. In puppies, the most common UI cause is unilateral or bilateral ectopic ureter, while in adults the most frequent non-neurogenic cause is urethral sphincter mechanism incompetence (USMI), of which anatomical and hormonal anomalies can play a role (5–8). This cause is seen in 60% of the total of UI affected dogs (9, 10). Frequently, USMI develops over time and owners delay visiting the veterinarian until the UI frequency or severity reaches an intolerable point. Up to 39% of dogs had signs of UTI 1–2 years before visiting the veterinarian (7). Incontinent dogs might present with intermittent or continuous UI with varying degrees of severity. Generally, it worsens when the animal is lying down or during periods of increased abdominal pressure (11). The prognosis of USMI is usually fair to good with long-term therapy (12).

Other non-neurogenic causes of acquired UI are secondary bladder atony, detrusor instability, and urovaginal fistula. In this sense, a vesicovaginal fistula has been described as causing UI in a female dog (13). Likewise, an infestation by *Mesocestoides vogae* caused UTI in a dog due to the presence of parasitic cysts around the bladder, which inhibited its function (14).

On the other hand, neurogenic causes such as spinal cord injury or contusion, often as a consequence of intense exercise or trauma, can lead to acquired UI (15, 16). Also, neoplasia such as spinal cord lymphoma is a cause of neurogenic UI (17). Neurogenic causes also include detrusor dyssynergia and primary bladder atony (12).

The consequences of UI are very unfavorable for those affected. Owners often show feelings of anger and frustration (7) and they even consider euthanasia for the affected animal (18, 19). In an American study about the causes of pet abandonment in shelters, soiling in the house was the only reason in 9.5% of abandonments and a very important factor in a set of causes in 18.5% of cases (20). Furthermore, the direct impact of UI on female dog welfare includes a higher risk of urinary tract infections and irritation due to continuous wetness of urine (21).

To date, there is no data on urinary incontinence in dogs in either Spain and Italy, or updated guidelines for its diagnosis and treatment. Therefore, the objective of this work is to collect information through an anonymous survey of veterinary professionals about the cases they see, the diagnosis and treatment of this condition, the follow-up veterinarians carry out on their cases and the interest they have in this subject. The collected information compares the UI situation between these two countries and develops specific, concrete, and useful recommendations for clinical practice with the aim of helping the veterinarians to better manage this condition.

## Materials and methods

The authors of this study designed a questionnaire composed of 27 questions in total (simple YES/NO, multiple choice, and rating scale questions) addressed to small animal veterinary clinical professionals from Spain and Italy. This questionnaire is available in Supplementary material in English version. The questions asked about respondents characteristics, case studies, diagnosis, treatment, patient follow-up and respondents’ UI update. The questionnaire was distributed along with a project cover letter. The Spanish Association of Small Animal Veterinarians (AVEPA), veterinary colleges from all over Spain, the Vetoquinol Italia mailing list and some closed Facebook groups collaborated in the distribution of the survey. The platform TYPEFORM SI (Barcelona, Spain) was used for the survey distribution. Participation in the survey was anonymous and voluntary.

Data were recorded in Microsoft Excel files and statistical analysis was carried out by using IBM SPSS Statistics 26.0 software (IBM Corp Armonk, NY, United States). Association between qualitative variants and comparisons between both countries were analyzed using Pearson’s chi square or Fishers’ exact test, alternatively. For multiple comparisons, Bonferroni correction was applied. *p*-values <0.05 were considered as significant.

## Results

In Spain, thanks to the collaboration of AVEPA, the questionnaire was made available to 5,350 veterinarians who are part of this organization; 305 responses were obtained (305/5,350;5.70%) In Italy 1,214 people viewed/opened the survey, and 971 started to fill it in (971/1214; 79.98%), while 552 completed it (552/1214; 45.47%).

The survey ended if the veterinarian indicated that he/she did not see any cases of UI within the previous three months (question 4). Thus, the analysis was restricted to 265 (Spain) and 462 (Italy) participants continuing the survey after question 4, which included questions on cases, diagnosis, treatment, follow-up, and interest in UI.

### Details about the respondents and their practice

Most of the respondents were women in both countries (72.5 and 69.6% in Spain and Italy, respectively), with 11 years or more of professional experience (69.2 and 75.5%, respectively) and worked in a small clinic (65.4 and 64.2%, respectively). In Spain, only 25.9% respondents worked in a referral clinic or hospital, 3.0% in a university, and the rest (5.75%) were self-employed veterinarians or corresponded to other categories. Similar percentages were found in Italy (29.5, 2.3 and 3.9%).

The possible association between gender and professional experience was analyzed. In Spain, both variables were associated (chisq = 34.992; df = 3; *p* < 0.001). Longer professional experience (>15 year) was more frequent in men than in women (77.8% vs. 41.3%), while both 5–10 years and < 5-year professional experience was more frequent in women (5–10 years: 24.8% vs. 7.4%; < 5 years: 14.7% vs. 1.2%). No significant difference was found between genders for 11–15 years of professional experience. Italy also showed a highly significant association between these variables (chisq = 11.536; df = 3; *p* = 0.009); however, longer professional experience (>15 year) corresponded to women (women: 68.9%; men: 54.6%) while 5–10 years of professional experience was more frequent in men (men: 18.1%; women: 9.0%).

In both Spain and Italy, most of the respondents lacked any specialization (30.4 and 42.6%, respectively). In Spain, the most frequent specializations were Internal Medicine (22.9%), Surgery (10.9%), Other (9.2%), Ultrasound/image diagnosis (4.1%), Emergency (3.8%), Dermatology (3.8%), and Oncology (3.8%). Less than 3% of responses corresponded to each of remaining specialization options available in the survey. In Italy, the specializations were somewhat different; the most frequent specializations were Surgery (12.2%), Other (11.3%), Internal Medicine (8.4%), Ultrasound/image diagnosis (5.7%), Anaesthesia (4.4%), and Reproduction (3.8%). For each of the remaining proposed specializations there were less than 3% responses.

In Spain, significant differences in respect to gender were found only for Cardiology (women: 0.0%; men: 7.0%; chisq = 7.03; df = 1; *p* = 0.008), Traumatology (women: 0.0%; men: 5.3%; chisq = 4.53; df = 1; *p* = 0.033) and Emergency (women: 7.6%; men: 0.0%; chisq = 4.61; df = 1; *p* = 0.032). In Italy significant differences were only found for Surgery (women: 8.4%; men: 20.1%; chisq = 14.81; df = 1; *p* < 0.001).

In Spain, non-specialized veterinarians mostly worked in small clinics (86.2% vs. 55.4% specialized veterinarians). On the contrary, most specialized veterinarians worked in referral clinics or hospitals (34.2% vs. 9.2% non-specialized veterinarians) and universities (4.5% vs. 0.0%) (chisq = 27.276; df = 4; *p* < 0.001). The situation was similar in Italy. Non-specialized veterinarians worked in small clinics (77.3% vs. 54.5% specialized veterinarians; chisq = 30.804; df = 1; *p* < 0.001). Specialized veterinarians worked in referral clinics or hospitals more frequently than non – specialized ones (39.2%vs. 17.6%; chisq = 29.93; df = 1; *p* < 0.001).

### Case studies

Table 1 shows the distribution of UI cases diagnosed per country, quarter, and gender.

**Table 1:** Distribution of UI cases diagnosed per country, quarter, and gender.

Variable	Category	Count/*n* (%)	
		Spain	Italy
UI cases per quarter			
	None	40/305 (13.1%)	90/552 (16.3%)
	≤3	194/305 (63.6%)	377/552 (68.3%)
	4–8	64/305 (21.0%)	75/552 (13.6%)
	9–15	6/305 (2.0%)	7/552 (1.3%)
	>15	1/305 (0.3%)	3/552 (0.5%)
Intact females showing UI			
	Unknown	13/263 (4.9%)	12/460 (2.6%)
	0–24%	231/263 (87.8%)	423/460 (91.9%)
	25–49%	14/263(5.3%)	16/460 (3.5%)
	50–74%	5/263 (1.9%)	4/460 (0.9%)
	75–100%	0/263 (0.0%)	5/460 (1.1%)
Spayed females showing UI			
	Unknown	10/264 (3.8%)^a^	4/460 (0.9%)^b^
	0–24%	120/264 (45.5%)^a^	32/460 (6.9%)^b^
	25–49%	58/264 (22.0%)^a^	58/460 (12.6%)^b^
	50–74%	34/264 (12.9%)^a^	125/460 (27.2%)^b^
	75–100%	42/264 (15.9%)^a^	241/460 (52.4%)^b^
Intact males showing UI			
	Unknown	12/264 (4.5%)^a^	11/461 (2.4%)^a^
	0–1%	86/264 (32.6%)^a^	191/461 (41.5%)^b^
	1–4%	85/264 (32.2%)^a^	163/461 (35.3%)^a^
	5–10%	52/264 (19.7%)^a^	71/461 (15.4%)^a^
	>10%	29/264(11.0%)^a^	25/461 (5.4%)^b^
Neutered males showing UI			
	Unknown	29/265 (10.9%)^a^	50/459 (10.9%)^a^
	0–24%	201/265 (75.8%)^a^	220/459 (47.9%)^b^
	25–49%	14/265 (5.3%)^a^	63/459 (13.8%)^b^
	50–74%	12/265 (4.5%)^a^	50/459 (10.9%)^b^
	75–100%	9/265 (3.4%)^a^	76/459 (16.5%)^b^

Significant differences are indicated with^a,b^ (*p* < 0.05).

Most veterinarians in both Spain and Italy claimed to see 1–3 UI cases per quarter (63.6 and 68.3%, respectively). Both countries showed similar percentages of veterinarians who did not observed any UI cases per quarter (Spain: 13.1%; Italy 16.3%) and who observed ≥9 UI cases per quarter (Spain: 2.3%; Italy: 1.8%) (chisq = 9.422; df = 4; *p* = 0.051). The survey discontinued when the respondent stated that no cases of UI were diagnosed per quarter; therefore, analysis was restricted to 265 participants in Spain and 462 participants in Italy.

The distribution of UI cases in intact females was similar in both countries (chisq = 8.613; df = 4; *p* = 0.072). Most veterinarians found that intact females suffering from UI accounted for 0–24% cases (Spain: 87.8%; Italy: 91.9%). However, both countries significantly differed for the distribution of spayed females showing UI (chisq = 207.694; df = 4; *p* < 0.001). Almost half of respondents observed 0–24% spayed females with UI in Spain (45.5%), while 52.4% Italian respondents found a much higher percentage (75–100%).

In both countries, the percentage of incontinent males was clearly lower than in females, but male distribution was significantly different between Spain and Italy (chisq = 15.201; df = 4; *p* = 0.004). The most frequent response was 0–1%, even though it was significantly less frequent in Spain (32.6%) than in Italy (41.5%). The second most frequent response (1–4%) showed a similar percentage in both countries (Spain: 32.2% and Italy: 35.3%). On the other hand, a higher percentage of males suffering UI (>10%) showed low frequencies, though this was most frequently reported in Spain (11.0%) than Italy (5.4%).

The distribution of neutered males showing UI significantly differed in both countries (chisq = 66.516; df = 4; *p* < 0.001). The highest frequency corresponded to the option of 0–24%, but the percentage of this response was higher in Spain (75.8%) than in Italy (47.9%). The remaining options showed less frequency in Spain than in Italy.

### Diagnosis

In both countries, most of the respondents used a diagnostic algorithm, although users of such an algorithm were more frequent in Spain (88.7% vs. 65.3%; chisq = 47.631; df = 1; *p* < 0.001). The distribution of users of the diagnostic algorithm by professional experience was similar in both countries, with a maximum for >15 years (chisq = 1.436; df = 3; *p* = 0.697). Most of the diagnostic algorithm users were specialized veterinarians in both countries, but the percentage of specialized users was higher in Spain (73.5% vs. 65.3%, chisq = 4.015; *p* = 0.045). Table 2 shows the distribution of the first step of diagnosis in both countries.

**Table 2:** Distribution of the first step of diagnosis in both countries.

Category	Count/*n* (%)	
	Spain	Italy
Patient reporting and history, urinalysis	37/263 (14.1%)	55/460 (12.0%)
Patient reporting and history, blood work and urinalysis	44/263 (16.7%)	64/469 (13.9%)
Patient reporting and history, blood work, urinalysis and abdominal ultrasound	181/263(68.8%)	328/460 (71.3%)
Patient reporting and history and antibiotic treatment for UI	0/263 (0.0%)	9/460 (2.0%)
Patient reporting and history, and symptomatic treatment for UI	0/263 (0.0%)	1/460 (0.2%)
Other	1/263 (0.4%)	3/460 (0.7%)

The distribution of the first step of diagnosis was similar in both countries (chisq = 7.563; df = 5; *p* = 0.182). Patient reporting and history was of great importance, and was considered as a key point for the subsequent orientation of diagnostic choices. Its average grade on a scale of 0–10 was 8.7 in Spain and 8.5 in Italy. Most of the respondents used a complete diagnostic algorithm (Spain: 68.8%; Italy: 71.3%); direct approaches to therapy were rare (only attested in Italy, with 2.0% of responses).

Both countries significantly differed in the use of urethral/bladder pressure measurement (chisp =12.598; df = 4; *p* = 0.013). Most respondents ignored this procedure, but this percentage was higher in Italy (55.9%) than in Spain (46.0%). Only 0.4% of Spanish veterinarians and 2.0% of Italian veterinarians used this procedure, however, 46.0 and 35.2% respondents in Spain and Italy, respectively, would like to use it.

In both Spain and Italy, most of the respondents (64.2 and 63.2%, respectively) typically investigated for anatomical abnormalities; of those, 28.8% Spanish respondents and 26.2% Italian respondents only did for young patients. No significant differences between both countries were detected for this activity (chisq = 5.239; df = 3; *p* = 0.155). In Spain, searching for anatomical abnormalities was associated with using a diagnostic algorithm (chisq =14.613; df = 3; *p* = 0.002). When using a diagnostic algorithm, the frequency of searching for anatomical abnormalities was 68%, while this frequency is only 34.5% when no diagnostic algorithm was used. On the contrary, in Italy the frequency of searching for anatomical abnormalities was higher when no diagnostic algorithm was used (69.6% vs. 48.4%; chisq = 21.659; df = 3; *p* < 0.0001). Specialized veterinarians in Italy tended to investigate anatomical abnormalities more frequently than non-specialized veterinarians (chisq = 31.38; df = 3; *p* < 0.0001), but this tendency was not detected in Spain. Table 3 shows preferences for diagnostic methods when anatomical abnormalities were suspected.

**Table 3:** Preferences for diagnostic methods when anatomical abnormalities were suspected.

Search for anatomical abnormalities	Count/*n* (%)	
	Spain	Italy
No preference (specialist advice)	75/247 (30.4%)^a^	209/458 (45.6%)^b^
Radiology with contrast medium	65/247 (26.3%)^a^	53/458 (11.6%)^b^
Radiology without contrast medium	2/247 (0.8%)^a^	4/458 (0.9%)^a^
Urethrocystoscopy	17/247 (6.9%)^a^	85/458 (18.6%)^b^
CT urography	85/247 (34.4%)^a^	104/458 (22.7%)^b^
Other	3/247 (1.1%)^a^	3/458 (0.7%)^a^

Significant differences are indicated with^a,b^ (*p* < 0.05).

Preferences for these diagnostic methods were different between Spain and Italy (chisq =54.047; df = 5; *p* < 0.001). No preference, since these cases were treated by a specialist and urethrocystoscopy was the more frequent in Italy than in Spain (45.6% vs. 30.4 and 18.6% vs. 6.95%, respectively). However, in Spain, Radiology with contrast medium and CT urography (26.3 and 34.4%, respectively) were more frequent than in Italy (11.6 and 22.7%, respectively). No significant differences were detected for Radiology without contrast medium and Other, and were very rare in both countries. In Spain “Other” referred to ultrasound and in Italy “Other” included ultrasound and combined methods.

When a UI patient showed reduced mobility from a possible neurological origin, the actions chosen by the respondents differed significantly between both countries (chisq = 85.773; df = 4; *p* < 0.001). No action while waiting for neurologist opinion is the most frequent option in Italy (55.3%); its frequency was only 23.6% in Spain. Drug UI treatment while waiting for specialist opinion was as frequent in Spain (22.8%) as in Italy (22.2%).

In Spain, no significant association was detected for these options and specialization (chisq =4.894; df = 4; *p* = 0. 298) or using a diagnostic algorithm (chisq =4.740; df = 4; *p* = 0. 315). In contrast, these options and specialization were associated in Italy (chisq = 18.04; df = 5; *p* = 0.0029); specialized veterinarians preferred no action while waiting for the specialist opinion (58.2% vs. 51.3% in non-specialized veterinarians). Also, these options and use of diagnostic algorithms were associated in Italy (chisq = 18.25; df = 5; *p* = 0. 0026). Thus, users of the diagnostic algorithm tended to proceed step by step; they did not proceed to treat if they did not have the specialist’s opinion (57.9% compared to 50.3% of non-users).

### Treatment

Table 4 shows the distribution of treatment in UI patients with suspected neurological origin.

**Table 4:** Distribution of treatment in UI patients with suspected neurological origin.

Treatments in UI patients with suspected neurological origin	Count/*n* (%)	
	Spain	Italy
Drugs promoting continence (DPC)	112/372 (30.1%)^a^	192/589 (32.6%)^a^
Drugs promoting bladder emptying (DPBE)	107/372 (28.8%)^a^	179/589 (30.4%)^a^
Muscle relaxant (MR)	51/372 (13.7%)^a^	46/589 (7.8%)^b^
Manual bladder emptying (MBE)	97/ 372 (26.1%)^a^	134/589 (22.8%)^a^
Other	5/372 (1.3%)^a^	38/589 (6.5%)^b^

Significant differences are indicated with^a,b^ (*p* < 0.05).

Since each respondent could choose more than one option, the totals were calculated for each treatment. Significant differences were detected between Spain and Italy for these treatments (chisq = 22.853; df = 4; *p* < 0.001). In both countries, the most frequent response was DPC, followed by DPBE and MBE, but the use of MR and Other significantly differed between countries. In Italy, “Other” referred to looking for a specialist opinion but this option was not detailed in Spain.

When blood and urine analyses were normal but urethral sphincter mechanism incompetence (USMI) was suspected, most of respondents started a pharmacological trial, although this percentage was higher in Spain (93.2%) than in Italy (78.9%) (chisq = 25.743; df = 1; *p* < 0.001).

In Spain, pharmacological trial was not associated with the veterinarian type of practice (chisq = 6.213; df = 4; *p* = 0.184) or specialization (chisq = 0.149; df = 1; *p* = 0.699). On the contrary, in Italy, respondents working in a small clinic used pharmacological trial more frequently than every other type of practice (chisq = 5.543; df = 1; *p* = 0.0186). Respondents from universities used pharmacological trial the least (Fisher’s exact test = 0.0085). Also, in Italy, the pharmacological trial was less frequent for specialized veterinarians (74.6%) than for non-specialized veterinarians (84.9%) (chisq = 6.85; df = 1; *p* = 0.0088).

In both Spain and Italy, pharmacological trial was associated with observed cases per quarter; pharmacological trial was less frequently used by respondents seeing more cases per quarter (Spain: chisq = 18.873; df = 3; *p* < 0.001; Italy: chisq = 9,74; df = 3; *p* = 0.0209). Also, only in Spain, pharmacological trial was more frequent for respondents using a diagnostic algorithm (94.5% vs. 83.35%; chisq = 5.210; df = 1; *p* = 0.022). It should be noted that the use of pharmacological trial was very frequent in Spain (90.1% of respondents used pharmacological trial); therefore, these high percentages of use in both diagnostic algorithm users and non-users are not surprising, even if a significant difference was found in favor of diagnostic algorithm users.

Respondents who answered “yes” to the question about pharmacological trial were asked to rank the following treatments in order of preference (see Table 5). The ranking of priority is a categorical variable whose values range from 1 to 7 (columns in Table 5). The cells in Table 5 show the count and percentage (count/n, %) per treatment/rank value of all responses in Spain and Italy, respectively. For example, in Spain, a total of 210 responses corresponded to rank of treatment; when reading by columns, most of them (170/210 = 81.0%) considered Phenylpropanolamine as the first treatment in order of preference, the other ones being considered as first treatment rarely. When reading by rows, Phenylpropanolamine was considered as the first treatment by most of responses (170/210 = 81.0%) and the second one by 32/210 (15.7%) responses, while the responses that positioned it lower in the ranking were very rare.

**Table 5:** Priority for several treatments.

Treatment	Priority													
	1		2		3		4		5		6		7	
	Spain	Italy	Spain	Italy	Spain	Italy	Spain	Italy	Spain	Italy	Spain	Italy	Spain	Italy
Phenylpropanolamine (median = 1 in both countries)	170/210 (81.0%)^a^	264/345 (76.5%)^a^	33/210 (15.7%)^a^	62/345 (18.0%)^a^	3/210 (1.4%)^a^	12/345 (3.5%)^a^	3/210 (1.4%)^a^	2/345 (0.6%)^a^	0/210 (0.0%)^a^	0/345 (0.0%)^a^	0/210 (0.0%)^a^	1/345 (0.3%)^a^	1/210 (0.5%)^a^	4/345 (1.2%)^a^
Ephedrine (median = 3 in both countries)	8/210 (3.8%)^a^	23/345 (6.7%)^a^	72/210 (34.3%)^a^	139/345 (40.3%)^a^	59/210 (28.1%)^a^	102/345 (29.6%)^a^	36/219 (17.1%)^a^	46/345 (13.3%)^a^	21/210 (10.0%)^a^	19/345 (5.5%)^a^	9/210 (4.3%)^a^	13/345 (3.8%)^a^	5/210 (2.4%)^a^	3/345 (0.9%)^a^
Deslorelin (median = 3 in both countries)	5/210 (2.4%)^a^	8/345 (2.3%)^a^	24/210 (11.4%)^a^	64/345 (18.6%)^b^	88/210 (41.9%)^a^	140/345 (40.6%)^a^	58/210 (27.6%)^a^	95/345 (27.5%)^a^	25/210 (11.9%)^a^	25/345 (7.2%)^a^	10/210 (4.8%)^a^	7/345 (2.0%)^a^	0/210 (0.0%)^a^	6/345 (1.7%)^a^
Estriol (median = 4 in both countries)	19/210 (9.0%)^a^	38/345 (11.0%)^a^	55/210 (26.2%)^a^	61/345 (17.7%)^a^	21/210 (20.0%)^a^	54/345 (15.7%)^a^	75/210 (35.7%)^a^	126/345 (36.5%)^a^	30/210 (14.3%)^a^	46/345 (13.3%)^a^	7/210 (3.3%)^a^	13/345 (3.8%)^a^	3/210 (1.4%)^a^	7/345 (2.0%)^a^
Collagen implant (median = 5 in both countries)	0/210 (0.0%)^a^	2/345 (0.6%)^a^	5/210 (2.4%)^a^	3/345 (0.9%)^a^	6/210 (2.9%)^a^	5/345 (1.4%)^a^	7/210 (3.3%)^a^	29/345 (8.4%)^a^	110/210 (52.4%)^a^	185/345 (53.6%)^a^	68/210 (32.4%)^a^	97/345 (28.1%)^a^	14/210 (6.7%)^a^	24/345 (7.0%)^a^
Drug association (median = 6 in both countries)	3/210 (1.4%)^a^	6/345 (1.7%)^a^	13/210 (6.2%)^a^	13/345 (3.8%)^a^	31/210 (14.8%)^a^	28/345 (8.1%)^a^	23/210 (11.0%)^a^	41/345 (11.9%)^a^	24/210 (11.4%)^a^	61/345 (17.7%)^a^	106/210 (50.5%)^a^	179/345 (51.9%)^a^	10/210 (4.8%)^a^	17/345 (4.9%)^a^
Other (median = 7in both countries)	5/210 (2.4%)^a^	4/345 (1.2%)^a^	8/210 (3.8%)^a^	3/345 (0.9%)^b^	2/210 (1.0%)^a^	4/345 (1.2%)^a^	8/210 (3.8%)^a^	6/345 (1.7%)^a^	0/210 (0.0%)^a^	9/345 (2.6%)^b^	10/210 (4.8%)^a^	35/345 (10.1%)^b^	177/210 (84.3%)^a^	284/345 (82.3%)^a^

Data are count/*n* (%). Significant differences are indicated with^a,b^ (*p* < 0.05).

Results showed a consensus of opinion on the priority of the treatments to be used. In both countries the median range attributed to each treatment was the same. The order of priorities was the same in both countries: Phenylpropanolamine, Ephedrine, Deslorelin, Estriol, Collagen implant, drug association and Other. The therapeutic preference in both countries corresponded to Phenylpropanolamine (Spain: 81.0%; Italy: 76.5%). Significant differences between countries were detected for Deslorelin (chisq = 14.214; df = 6; *p* = 0.027); range 2 was more frequent in Italy than in Spain (Spain: 11.4%; Italy: 18.6%). Also, both countries differed for Other (chisq = 19.368; df = 6; *p* = 0.004), in ranges 2 (Spain: 3.8%; Italy: 0.9%), 5 (Spain: 0.0%; Italy: 2.6%) and 6 (Spain: 4.8%; Italy: 10.1%).

In the case of USMI, when pharmacotherapy failed, the most frequent option was drug change, followed by an increase in the dosage/frequency of administration of the drug in both Spain and Italy (see Table 6). Colposuspension was the third option although chosen much less frequently than the previous two options. Only 1.3% of Spanish respondents chose to send the case to a specialist, versus 4.5% Italian respondents; this fact caused a significant difference between countries for this variable (chisq = 19.304; df = 10; *p* = 0.037). The remaining options (More advanced diagnostics, Homeopathy/acupuncture and Never happened) were only chosen in Italy.

**Table 6:** Therapeutic options when pharmacotherapy failed.

Therapeutic options when pharmacotherapy failed	Count/*n* (%)	
	Spain	Italy
Bulking agents/collagen implant	5/239 (2.1%)^a^	19/448 (4.2%)^a^
Increased drug dosage/frequency of administration	94/239 (39.4%)^a^	162/448 (36.2%) a
Drug change	116/239 (48.5%)^a^	184/448 (41.1%)^a^
Colposuspension	14/239 (5.9%)^a^	35/448 (7.8%)^a^
Reconstructive surgery of the urinary bladder neck	2/239 (0.8%)^a^	1/448 (0.2%)^a^
Artificial sphincter	3/239 (1.3%)^a^	8/448 (1.8%)^a^
Urethral sling	2/239 (0.8%)^a^	5/448 (1.1%)^a^
Send to specialist	3/239 (1.3%)^a^	20/448 (4.5%)^b^
More advanced diagnosis	0/239 (0.0%)^a^	4/448 (0.9%)^a^
Homeopathy/acupuncture	0/239 (0.0%)^a^	7/448 (1.6%)^a^
Never happened	0/239 (0.0%)^a^	3/448 (0.7%)^a^

Significant differences are indicated with^a,b^ (*p* < 0.05).

In Spain, non-specialized veterinarians chose drug change more frequently than specialized veterinarians (62.1% vs. 42.3%; chisq = 7.371; df = 1; *p* = 0.007). In Italy, colposuspension was chosen more frequently by specialized veterinarians than by non–specialized veterinarians (11.6% vs. 2.7%; chisq = 11.720; df = 1; *p* = 0.001). No other associations were detected in both countries.

After surgical correction, most respondents in Spain (53.3%) and a high proportion in Italy (43.5%) did not know how many individuals also required medical treatment. Medical treatment associated with surgical correction occurred in 0–24% UI cases (Spain: 35.5%; Italy: 45.2%). Higher percentages for this association were chosen with frequency > 10% (25–49%) and even <3% (50–100%) in both countries. No significant differences were found between the two countries for this therapeutic association (chisq = 7.490; df = 4; *p* = 0.112).

In both countries when an UI patient had normal blood and urine tests but showed an anatomical abnormality, most respondents did not act but waited for an imaging diagnosis (Spain: 62.7%; Italy: 65.0%). However, in this situation, 36.5% Spanish respondents and 30.6% Italian respondents began pharmacological treatment while waiting for an imaging diagnosis. Significant differences between countries were only found in the Other options (Spain: 0.8%; Italy: 4.4%; chisq = 8.667; df = 2; *p* = 0.013).

There were significant differences between countries for actions taken when UI was not completely solved (chisq =197.762; df = 13; *p* < 0.001). Table 7 shows these actions in both countries. Most veterinarians from both countries consulted a specialist; specialist type was different according to the country. Furthermore, more advanced diagnosis was more frequent in Spain than Italy (10.6% vs. 6.5%, respectively) as well as the fact that the respondent was no longer the veterinarian in charge of the case (dropped case; 12.2% vs. 0.0%, respectively). Finally, a small percentage, similar for both countries, did not act considering that the UI was never fully resolved (Spain: 1.2%; Italy: 3.1%).

**Table 7:** Actions taken when UI was not completely resolved.

Action	Count/*n* (%)	
	Spain	Italy
No action	3/247 (1.2%)^a^	14/452 (3.1%) a
Neurologist consultation	71/247 (28.7%)^a^	112/452 (24.8%)^a^
Internal medicine consultation	32/247 (13.0%)^a^	40/452 (8.8%)^a^
Surgery consultation	11/247 (4.5%)^a^	44/452 (9.7%)^b^
Reproductive medicine consultation	6/247 (2.4%)^a^	28/452 (6.2%)^b^
Diagnosis by image consultation	20/247 (8.1%)^a^	14/452 (3.15)^b^
Other specialists’ consultation	8/247 (3.2%)^a^	0/454 (0.0%)^b^
Unspecified specialist consultation	53/247 (21.5%)^a^	0/454 (0.0%)^b^
Change a drug	9/247 (3.6%)^a^	9/454 (2.0%)^a^
Increased dose/frequency of the used drug	5/247 (2.0%)^a^	12/454 (2.7%)^a^
A diagnostic test is added	16/247 (6.5%)^a^	93/454 (10.6%)^b^
A new drug is added	13/247 (5.3%)^a^	16/454 (3.5%)^a^
Dropped case	0/454 (0.0%)^a^	55/454 (12.2%)^b^
Other	0/454 (0.0%)^a^	15/454 (3.5%)^b^

Significant differences are indicated with^a,b^ (*p* < 0.05).

### Patient follow-up

Patient follow–up after medical/surgical treatment significantly differed between countries (chisq =20.470; df = 3; *p* < 0.001). Re-evaluation after one week was the most frequent option, even though this frequency was significantly higher in Spain (58.0%) than in Italy (41.4%). With the exception of the Other option (low frequency in both countries), the re-evaluation after 3 weeks was the least frequent chosen option, with this frequency being significantly higher in Italy (18.8%) than in Spain (10.4%). Re-evaluation after 2 weeks did not show significant differences between countries (Spain: 31.2%; Italy: 38.5%).

Timing of re-evaluation also differed between countries (chisq =4.664; df = 1; *p* = 0.031). Most veterinarians recommended re-evaluation at regular intervals, but this option was more frequent in Italy (78.2%) than in Spain (71.0%). The re-evaluation only when UI reoccurs was advised for only 29.0% respondents in Spain and 21.8% in Italy. In Italy, the re-evaluation at regular intervals was associated with certain characteristics of respondents: this timing was a more typical behavior of those who follow a diagnostic algorithm (82.7%) (chisq = 10.32; df = 1; *p* = 0.0013) and of those who had a specialization (81.8%) (chisq = 4.87, df = 1; 0 = 0. 0273). On the contrary, these associations were not detected in Spain.

In both countries, the patient follow-up lasted between several months and a year; in total, these two options exceeded 60% of the responses obtained in both countries. Longer follow-up (>5 years) was rare (Spain: 8.1%; Italy: 11.0%). No significant differences were found between countries (chisq = 8.84; df = 5; *p* = 0.116). In Italy, more professional experience (>10 years) was significantly associated with the ability to maintain longer follow-up (>3 years) (chisq = 9.88, df = 1; *p* = 0.0016). However, this association did not occur in Spain (chisq = 0.082, df = 1; *p* = 0.775).

### UI update

No significant differences were found between countries (chisq = 3.994, df = 2; *p* = 0.136). Most of respondents were current on UI updates only if UI occurred in their common practice (Spain: 63.0%; Italy: 55.4%). About 30% of respondents were current on UI (Spain: 30.5%; Italy: 37.4%) and the rest were not current at all (Spain: 6.5%; Italy: 7.2%). In Italy, those staying up to date on UI were associated with the use of a diagnostic algorithm (chisq = 8.158; df = 2; *p* = 0.0169) and with a higher frequency of UI cases/quarter. Veterinarians who saw ≥4 cases per quarter were usually up to date on UI information, while those with fewer cases mostly chose “updated only if UI occurred “(chisq = 9.312; df = 2; *p* = 0.009).

In Spain, staying up to date was also associated with using a diagnostic algorithm (chisq =12.027; df = 2; *p* = 0.002). In respect to the frequency of UI cases/quarter, no significant association was detected, but *p* value was very near the limit for significance (chisq =5.858; df = 2; *p* = 0.053), suggesting a tendency similar to Italy’s.

## Discussion

This study was based on a questionnaire administered online and the participation of the veterinarians in the survey was voluntary; therefore, participation bias would appear if responders differed for result variables. Since respondents were anonymous, it was not possible to assess whether any differences between respondents and non-respondents would affect their responses. With the help of the associations of clinical veterinarians of both countries, an attempt was made to reach the largest number of veterinary doctors; the greater the number of responses, the greater the representativeness of the sample analyzed and the fewer problems associated with participation bias. Nonetheless, the participation of veterinarians in this survey could be considered as moderate and this is one of the limitations of this study.

### Details about the respondents and their practice

In both countries, most of the respondents were women; several authors suggested that women were more likely to participate in this type of survey (22–24). The greater number of years of practice for the majority of respondents from both countries could reflect the greater attention to the UI issue. A great percentage of veterinarians worked in small clinics in both countries, suggesting great interest in UI by primary care veterinarians, most of them without particular specialization.

### Case studies

The obtained results suggested that UI was not very frequently observed, although a greater frequency was observed in spayed females. According to the literature, estimated prevalence in spayed females was 3–5% even though several initial studies found 30% prevalence and even 40% in large breeds (25–28). However, in intact females, prevalence was only about 1% (4, 26–28).

Patients affected by UI are typically young or middle-aged spayed females (27). In fact, previous spaying was associated with increased likelihood (odds) of UI in females (29). In both USA and Europe neutering male and female dogs became routine and performing it at six months of age and even earlier is increasingly common (30). There is no consistent evidence of relationship of age at ovariohysterectomy and UI development in female dogs (29, 31, 32). In most cases, UI developed within three years following spaying, although clinical signs could be observed immediately or delayed up to 10 years (27).

However, a relationship between the weight of females at UI diagnosis and the age at spaying would affect the probability of developing it (33). Spaying females of expected adult weight > 25 kg later in their first year of life would decrease this probability; for females under this expected adult weight, age at spaying may have no impact on UI probability. Furthermore, UI occurred within a few years after spaying in heavier females (28).

According to this, it is necessary to evaluate the need for spaying on a case-by-case basis, considering the age of the female, the breed predisposition to UI and the desire to prevent hormonally mediated tumors. In general, it would not be advisable to spay before the first heat (31).

The percentage of incontinent males was clearly lesser than in females, as seen in both countries, and it was in accordance with previous results (28). In males, UI followed a pattern of presentation and signs similar to females, so heavier, middle-aged and neutered males were more frequently affected by UI (3). For most of the cases seen by veterinarians in both countries only 0–24% of incontinent males were neutered while data in the literature stated that most of incontinent males were neutered (28).

Perhaps the prevalence of UI was underestimated in this work; its presence could be underestimated by the owner, especially in animals that live outdoors. Therefore, it is important to educate the owner on the topic and ask the right questions during the clinical visit.

### Diagnosis

The survey revealed that most veterinarians in both countries used a diagnostic algorithm, especially those who have some specialization. These results were encouraging, but raising everyone’s awareness of its use is important, especially in Italy, with a smaller percentage of users. Following a step-by-step approach is essential, as in human medicine (34). A diagnostic/therapeutic algorithm has been published in Italy (35) and Spain (36).

Ideally, UI diagnosis would start with a complete medical history (anamnesis), physical and neurological examination, urinalysis (test strips and sedimentation) and urine culture (11, 37). Medical history must include age, breed, sex, clinical signs (start and progression), water intake, frequency of urination, volume and color of urine, and discomfort or difficulty when urinating. A history of intermittent incontinence, particularly when the patient is relaxed or resting, would point to USMI.

Anamnesis should be based on forms filled out by the owner(s), designed to better characterize incontinence problems and to objectively monitor the situation over time. In the physical examination, there are no specific signs pointing to USMI, but special attention should be paid to size, tone and location of the bladder, to prostatic and urethral palpation by rectal examination and to the examination of the external genitalia. Urinalysis and urine culture allow testing for concurrent polyuria/polydipsia or urinary tract infections that may contribute to the severity of UI.

Upon the results, the subsequent tests should include hematology and serum biochemistry testing (evaluation of kidney function and detection of co-occurrence/exacerbation of metabolic diseases) and abdominal ultrasonography and/or radiography (detection of anatomic abnormalities or acquired defects). Abdominal ultrasonography shows 50% sensitivity for diagnosis of congenital defects leading to UI.

Abdominal ultrasonography and radiography are usually normal in USMI patients; however, these diagnostic imaging methods of the abdominal area should help rule out the presence of stones in the urinary tract or concurrent neoplasms. These results point to a general sensitivity of veterinarians towards the search (and eventually correction) of UI causes or predisposing factors, not limited solely to symptomatic treatment.

In the differential diagnosis of USMI with respect to other forms of UI, measuring urethral/bladder pressure is important, since it allows the evaluation of the sphincter contraction force (38). However, this test requires specialized equipment and usually only few universities and referral centers have them available (39). This fact would explain the limited knowledge and use of this technique by respondents, even though specialized veterinarians were more interested in knowing and using it. However, a presumptive diagnosis is often made regarding the response to a specific USMI treatment (3, 37).

Most responders from both countries did not know about or use the urethral bladder pressure measurement. This fact would be explained by a lack of standardization, application problems and variability in the interpretation of results of this test, thus justifying the diagnosis by exclusion of USMI. It is remarkably interesting that 46.0% of veterinarians in Spain and 35.2% in Italy would like to use this technique. This measure is also useful for monitoring before and after surgery for ectopic ureter and other anatomical anomalies or in relation to medical therapy (39–42).

It is encouraging that most responders in both countries went in search of anatomical abnormalities; surprisingly, however, almost a quarter of veterinarians believe that anatomical abnormalities are unique to young animals (they only look for them in young animals). According to the clinical situation of the UI patient, it would be possible to evaluate whether to try a drug while waiting for the results of imaging diagnosis. However, it is necessary to investigate/exclude anatomical abnormalities even in mature patients (43), since many different abnormalities in addition to the ectopic ureter can cause incontinence.

In both countries, CT urography was the preferred diagnostic method when anatomical abnormalities were suspected. CT urography has 90% diagnosis sensitivity and, on some occasions, may recognize the intramural and extramural ectopic ureter; this differentiation is very important for surgical approach. On the other hand, urethrocystoscopy could reach 100% diagnostic sensitivity and allows evaluation of the entire lower urinary tract from the vestibule to the bladder. In this way, urethrocystoscopy could diagnose abnormalities in vestibule/vagina (e.g., persistent mesonephros) that are difficult to diagnose by other image diagnosis techniques. However, these diagnostic techniques used to be complementary; the urethrocystoscopy by itself, without CT urography or ultrasound assessment, cannot give information about ureter dilatation, kidney status and other regions (44–46).

### Treatment

When facing an UI case with suspected neurological origin, most respondents in both countries used drugs promoting continence and promoting bladder emptying, even while waiting for the results of definite diagnosis. Lower percentages used manual bladder empty. These options largely reflect current literature, which focuses on the mechanism and usefulness of drugs to the detriment of manual bladder management.

Both in cases of upper and lower motor neuron urinary incontinence (hence, in both defects of storage and emptying), manual bladder emptying could really be effective in daily patient management at home and in the hospital. Furthermore, this technique minimizes the permanent damage risk by bladder distention pending resolution of the primary neurological disease, when possible. Unfortunately, this subject is rarely studied in literature; the only studies about manual bladder emptying deal with differences between intermittent or permanent catheterization and manual bladder emptying in terms of secondary risk of infections in the urinary tract (47). Nowadays, there is not clear data nor guidelines on proper bladder management for patients with incontinence of neurological origin. These guidelines should include in most cases manual bladder emptying as the main tool with the help of drug therapy in more complex cases, and not in the reverse order (48).

Under USMI suspicion with otherwise a normal exam, most of the respondents started a pharmacological treatment. This fact pointed to a hormonal basis for USMI, in line with current literature (35). Use of pharmacological treatment was inversely associated with cases observed per quarter; this fact could be because those who see more cases tend to be second-opinion veterinarians when the pharmacological treatment has already been carried out.

For pharmacological treatment, respondents from both countries clearly preferred Phenylpropanolamine (PPA); estrogens ranked fourth in therapeutic preference. Phenylpropanolamine, as a sympathomimetic drug, represents a quick and effective solution and the first-choice treatment in case of USMI, due to its actions on the contraction of the urethral sphincter and the smooth muscle of the urethra. Mean efficacy of PPA is >75% and if the therapy is carried out continuously, continence will be reached in 95% of patients. Furthermore, continence is recovered in 72 h, in some females even in 24 h (49–51).

When pharmacotherapy failed, the percentage of Italian respondents who immediately recommended the use of bulking agents/collagen implant seemed to be too high (4.2%), especially when compared with a very low frequency of respondents who preferred reconstructive surgery of the urinary bladder neck (0.2%). An analogous situation was found in Spain, where these percentages were 2.1 and 0.8%, respectively. In this sense, it must be considered that the intraurethral injection of bulking agents is not a definite solution for incontinence; the effect lasts 6–18 months, depending on its association with medical treatment (8, 52, 53). Before using a surgical procedure that is more aggressive and requires anesthesia, trying a combination of drugs, changing drugs or increasing drug dosage/frequency of administration would be more appropriate; most respondents in both countries chose these options.

Furthermore, for a conservative approach to UI treatment, there are a series of tips for the owner, similar to ones for human patients: regular and moderate physical activity, weight loss in overweight or obese patients, keeping an incontinence diary (identification of an incontinence pattern and planning for the animal to urinate just before the identified times) (34, 54).

Surprisingly, more than a third of respondents in both countries increased the dose/frequency of administration; this fact suggests that it is not always customary to start with a full dose and in accordance with the indicated dose. It would be recommended to start with a full dosage for PPA (1–1.5 mg/kg three times a day) (17, 55) and then find the minimum effective dose once the patient is stabilized; in fact, many patients maintained urinary continence with only a single daily dose of PPA (49–51, 55, 56).

In both countries, a high proportion of respondents did not know how many patients needed medical treatment after surgical correction. This fact points to poor perception and consideration of a frequent persistence of UI even after surgical treatment (e.g., due to anatomical abnormalities) and therefore an additional medical treatment is usually needed. In fact, UI is a multifactorial process and to proceed step-by-step is necessary. Furthermore, the option mostly chosen when medical treatment was associated with surgical correction was 0–24%, a very low percentage when compared with literature (10, 57–61), which confirms the need for awareness on this point.

Another explanation for these low percentages of medical treatment associated with surgical correction would be a high success rate of surgery as a treatment of UI. For the surgical correction of ectopic ureters in the dog female, the percentage of post-surgical continence ranged from 42 to 71% with traditional surgery, and this percentage increased with the addition of medical therapy according to previous studies (55–59). With endoscopic correction with laser, the continence obtained varies from 31 to 47% without medical therapy and increases from 69 to 77% with the addition of medical therapy (59, 62–64).

### Patient follow-up

Lack of data about how many patients needed medical treatment after surgical correction would be explained by a short follow up; most respondents stated that their follow-up of the patients was no longer than one year. Hence, subjects who, after surgery, experienced a long-term flare of clinical signs may not have been considered. A longer-term follow-up could clarify whether the need for post-surgical corrective medical treatment is really low, or appears later in time and, therefore is underestimated by veterinarians. The re-evaluation only when UI recurs was frequent in both countries; this finding is understandable in case of adjustment of medical therapy. However, the re-evaluation at regular intervals would be advisable to act promptly if necessary. The duration of patient follow-up seems to be related with customer loyalty, higher for those respondents having more years of working experience. For customer loyalty, it is important to clarify with the owner that complete recovery may be difficult, although it is possible to improve the patient’s condition, guaranteeing the pet and owner a better quality of life.

### UI update

Considering the percentages of veterinarians who actively or passively stay up to date on UI, interest in the topic is evident. The high percentage of colleagues who are interested in being updated on the topic “if it happens” (higher in Spain than in Italy) may suggest the need for greater awareness on the topic.

## Conclusion

It is worth highlighting the need for awareness about UI: this condition is often underestimated and requires increased awareness for adequate management. Accurate medical history and exam findings are essential for a correct UI diagnosis and effective treatment. A step-by-step diagnostic approach, with the use of blood-biochemical tests, abdominal ultrasound, and in some cases uroCT or urethrocystoscopy, is essential for an accurate diagnosis. Regular follow-up is important to evaluate the effectiveness of the therapy. Phenylpropanolamine is the drug of first choice for UI due to urethral sphincter mechanism incompetence. Manual bladder compression can be useful for patients with neurological UI.

## Data availability statement

The raw data supporting the conclusions of this article will be made available by the authors, without undue reservation.

## Author contributions

MF: Conceptualization, Investigation, Supervision, Visualization, Writing – original draft, Writing – review & editing. RC: Conceptualization, Investigation, Supervision, Visualization, Writing – original draft, Writing – review & editing. AG: Methodology, Writing – original draft, Writing – review & editing. MP: Investigation, Supervision, Visualization, Writing – original draft, Writing – review & editing. MT: Data curation, Methodology, Validation, Writing – original draft, Writing – review & editing. PT: Data curation, Methodology, Writing – original draft, Writing – review & editing. SN: Conceptualization, Investigation, Supervision, Visualization, Writing – original draft, Writing – review & editing. PZ: Investigation, Supervision, Visualization, Writing – original draft, Writing – review & editing. IL: Investigation, Supervision, Visualization, Writing – original draft, Writing – review & editing. EG-P: Conceptualization, Funding acquisition, Project administration, Resources, Visualization, Writing – original draft, Writing – review & editing. JR: Conceptualization, Funding acquisition, Project administration, Resources, Visualization, Writing – original draft, Writing – review & editing. DK: Methodology, Writing – original draft, Writing – review & editing. OM: Investigation, Supervision, Visualization, Writing – original draft, Writing – review & editing.
