# Supplementary material for: An international survey on canine urinary incontinence: case frequency, diagnosis, treatment and follow-up
Source: Front Vet Sci. 2024 Jul 17;11:1360288. doi: 10.3389/fvets.2024.1360288 (PMC11288968; doi:10.3389/fvets.2024.1360288)
Supplement: Supplementary file 1 [file Table_1.DOCX]

**SURVERY ON URINARY INCONTINENCE IN SMALL ANIMALS**

**First, a few questions about you:**

1. I have been a DVM/BVSc for:
   1. Less than 5 years
   2. Between 5 and 10 years
   3. Between 11 and 15 years
   4. More than 15 years
2. In which aspect of veterinary medicine do you practice?
   1. Small Animal Practice
   2. Emergency and/or Referral Centre
   3. University
   4. Locum
   5. Other
3. What kind of speciality do you have?
   1. None
   2. Reproduction
   3. Anaesthesia
   4. Neurology
   5. Cardiology
   6. Radiology
   7. Internal Medicine
   8. Emergency/Critical Care
   9. Surgery
   10. Dermatology
   11. Oncology
   12. Sports Medicine and Rehabilitation
   13. Other
4. How many cases of UI do you usually see in a 3 month period?
   1. None *(the survey stops here)* Thank you for your participation
   2. ≤ 3
   3. between 4 and 8
   4. between 9 and 15
   5. More than 15

**Next, some questions about your experience with diagnosing Urinary Incontinence (UI):**

Of the dogs you have diagnosed with UI, what percentage are intact female dogs?

- 1. 0-24%
  2. 25-49%
  3. 50-74%
  4. 75-100%
  5. I don’t know

Of the dogs you have diagnosed with UI, what percentage are spayed female dogs?

- 1. 0-24%
  2. 25-49%
  3. 50-74%
  4. 75-100%
  5. I don’t know

Of the dogs you have diagnosed with UI, what percentage are intact male dogs?

- 1. ≤1%
  2. 1-4%
  3. 5-10%
  4. More than 10%
  5. I don’t know

Of the male dogs you have diagnosed with UI, what percentage are neutered male dogs?

- 1. 0-24%
  2. 25-49%
  3. 50-74%
  4. 75-100%
  5. I don’t know

1. Do you routinely use a diagnostic algorithm when you suspect a case of UI?
   1. Yes
   2. No
   3. Other
2. If you answered “Other” to the previous question, would you like to explain?
3. In a dog with UI what do you think the first important step is?
   1. Patient reporting and history, urinalysis
   2. Patient reporting and history, blood work and urinalysis
   3. Patient reporting and history, blood work, urinalysis and abdominal ultrasound
   4. Patient reporting and history and antibiotic treatment for urinary incontinence
   5. Patient reporting and history and symptomatic treatment for UI
   6. Other
4. On a scale of 1 to 10, how important are patient reporting and history in your initial diagnostic choices?  1= not important) and 10=very important
5. Do you use systems for measuring urethral/bladder pressure in patients with UI?
6. Yes
7. No, I’m not interested in this measurement
8. No, but I would like to learn more about this diagnostic test
9. No, I believe the diagnosis for urinary incontinence is based on clinical signs
10. I don’t know what these diagnostics test are
11. Other
12. Do you usually investigate for anatomic abnormalities in patients with UI?
    1. Yes
    2. No
    3. Only in young patients
    4. Other
13. For a case with a suspected anatomic abnormality your favorite advanced diagnostic test is:
    1. uroTC
    2. Urethrocystoscopy
    3. Radiology with contrast medium
    4. Radiology without contrast medium
    5. I have no preference, the specialist takes care of it
    6. Other
14. If a dog with UI has normal blood work and urinalysis results but you suspect an anatomic anomaly and have ordered advanced diagnostic imaging, you would….
    1. Start medical treatment for UI while waiting for results of the diagnostic imaging
    2. You don’t do anything while waiting for results of the diagnostic imaging
    3. Other

**Next, some questions about your experience with treatment of Urinary Incontinence (UI):**

If the preliminary diagnostic testing results are normal, and you suspect Urethral Sphincter Mechanism Incompetence (USMI), do you usually start with a pharmacological trial?

- 1. Yes
  2. No

If you answered yes to question 12 above, please put the following therapeutic options in order of preference:

- 1. Phenylpropanolamine
  2. Ephedrine
  3. Association of different drugs
  4. Collagen implant
  5. Other

If there is no therapeutic response to the first choice drug you used for USMI, you would next suggest:

- 1. Colposuspension
  2. Urethral Sling
  3. Artificial sphincter
  4. Bulking agents/Collagen implant
  5. Reconstructive surgery of the urinary bladder neck
  6. A change of drug used
  7. An increase of drug dosage and/or frequence of administration
  8. Other

Can you indicate, in your experience, the percentage of cases in which it is necessary to initiate medical management following surgical correction for UI or USMI?

- 1. 0-24%
  2. 25-49%
  3. 50-74%
  4. 75-100%
  5. I don’t know

1. What do you do if after medical and/or surgical treatment a patient’s UI doesn’t completely resolve?
   1. I contact a specialist (chose one from below)
      1. Neurology
      2. Internal medicine
      3. Surgery
      4. Theriogenology
      5. Radiologist
   2. I add a drug to the current treatment protocol
   3. I change the drug
   4. I increase the dosage/frequency of the drug used
   5. I further explore with other diagnostic testing
   6. I don’t do anything because it is never possible to resolve the UI
   7. Discuss humane euthanasia with the pet owner
2. What are your next steps for a patient with UI who also has impaired mobility of suspected neurological origin?
   1. Treat with a drug for UI and monitor clinical signs
   2. Treat with a drug for UI while waiting for a referral appointment with a Neurologist
   3. You don’t do anything while waiting for a referral appointment with a Neurologist
   4. Other
3. For a case with a diagnosis of Neurologic UI which type of treatment(s) do you take in to consideration? (multiple answers possible)
   1. Drugs that promote the bladder emptying
   2. Drugs that promote continence
   3. Muscle relaxant
   4. Manual emptying of the bladder
   5. Other

**Lastly, some questions about your experience regarding follow-up with Urinary Incontinence (UI) patients:**

1. In case of medical or surgical treatment, after how long do you re-evaluate the patient?
   1. After 1 week
   2. After 2 weeks
   3. After 3 weeks
   4. Only if the symptoms of UI recur
   5. Other
2. On average how long are you able to follow your patients treated for UI?
   1. For a few months
   2. 1 year
   3. 2 years
   4. 3 years
   5. 4-5 years
   6. More than 5 years
3. Do you keep up with continuing education related to UI?
   1. Yes
   2. No
   3. If it happens
4. Please, feel free to leave any comment you may have about this subject

Thank you for participating in this survey. This study is made possible by Propalin® - Vetoquinol.
